# Deciphering the immunomodulatory cross-talk: Bacterial extracellular vesicles in gut homeostasis

**DOI:** 10.1080/21505594.2025.2566255

**Published:** 2025-09-30

**Authors:** Hui Yi, Mei Li, Le Xu, Huajie Mao

**Affiliations:** Department of Clinical Laboratory, The People’s Hospital of Wenjiang, Chengdu, China

**Keywords:** Bacteria, extracellular vesicles, immunity, intestinal tract

## Abstract

Within mammalian gastrointestinal ecosystems, trillions of microorganisms generate sophisticated ecological networks through bacterial extracellular vesicles (bEVs). Emerging evidence positions these bEVs as pivotal mediators in gut homeostasis maintenance and host-microbiota crosstalk, capable of transporting bioactive cargo including virulence determinants, genetic transfer components, and host-derived defense molecules. This molecular payload enables bEVs to exert multifaceted immunomodulatory effects through: 1) Immune cell activation and differentiation. 2) Microbial community regulation. 3) Epithelial barrier reinforcement. This review systematically examines current understanding of bEV biology: First, we characterize the structural complexity and compositional diversity of bEVs across bacterial species. Second, we elucidate the molecular machinery governing bEV biogenesis and secretion pathways. Third, we analyze mechanisms underlying bEV-immune interactions through receptor-mediated signaling and cargo delivery processes. By integrating recent advances in bEV immunobiology with mechanistic insights into host-pathobiont communication networks, our framework not only clarifies current knowledge gaps but also proposes standardized methodologies for future investigations.

## Introduction

The gut, a vital organ with dual functions of digestion/absorption and intestinal defense, hosts a dynamic microbial ecosystem comprising thousands of bacterial species and over 10 trillion microorganisms [1,2]. This complex community plays a pivotal role in human health and disease by shaping metabolic processes, maintaining epithelial barrier integrity, and modulating immune responses [3,4]. Despite lacking direct cellular connections or stable extracellular matrices, gut bacteria interact with host cells through diverse mechanisms [5]. Recent studies highlight bacterial extracellular vesicles (bEVs) as key mediators of this cross-kingdom communication [6]. These nanoscale particles traverse the gut vascular barrier (GVB) into systemic circulation, leveraging their surface structures to bind host or bacterial cells and deliver bioactive cargo, thereby influencing microbial viability and host physiology within the intestinal microenvironment [7]. Critically, how bEVs traverse the GVB to exert systemic effects remains poorly defined, limiting clinical translation of bEV research.

bEVs are spherical, bilayer lipid membrane-enclosed nanoparticles (20–350 nm in diameter) secreted by both pathogenic and commensal gut bacteria [8,9]. Packed with proteins, nucleic acids, and lipids, bEVs serve as versatile signaling vehicles that facilitate nutrient acquisition, antibiotic resistance, microbial competition, and host-microbe crosstalk [10]. Their ability to penetrate mucosal barriers and interact with diverse host cell types positions them as potent immunomodulators [11]. Emerging evidence underscores their roles in horizontal gene transfer, biofilm formation, and the delivery of virulence factors or toxins that may compromise epithelial integrity, trigger inflammation, or promote carcinogenesis [12–16]. Conversely, bEVs derived from beneficial symbionts can enhance host defense mechanisms and support immune homeostasis [17–19].

This review synthesizes recent advances in bEV biology, focusing on their biogenesis, immunoregulatory functions, and interactions with host immune cells. We further evaluate the therapeutic potential of bEVs in modulating intestinal immunity and address current challenges in translating bEV research into clinical applications, including limitations in mechanistic understanding and methodological standardization.

## Classifications and biogenesis of bEVs

**Figure 1.**
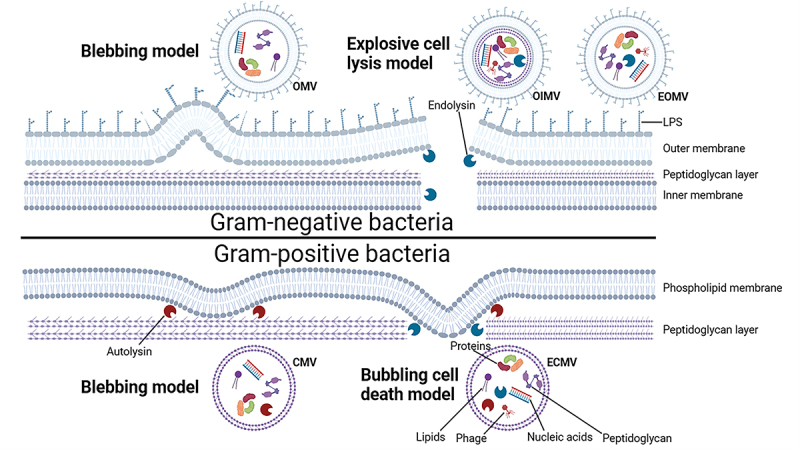
Biogenesis models and types of bEVs. Gram-negative bacteria can release outer membrane vesicles (OMVs) by blebbing of the outer membrane and explosive outer membrane vesicles (EOMVs) and outer-inner membrane vesicles (OIMVs) by explosive cell lysis produce, respectively (above panel). In the explosive cell lysis pathway, phage-encoded endolysins are produced and destroy the bacterial envelope. Gram-positive bacteria can secretory cytoplasmic vesicles (CMVs) by blebbing of the outer membrane and explosive CMVs (ECMVs) via “bubbling cell death,” respectively (below panel). Bubbling cell death refers to endolysin creating holes in the cell wall through which the bacterial membrane can bulge, leading to the release of bEV.

The intestinal microbiota exhibits profound metabolic activity, with emerging evidence highlighting fecal bacterial extracellular vesicles (bEVs) as critical mediators of intercellular communication and energy exchange within the gut microenvironment. Bacteria can be classified as gram-negative or gram-positive according to gram staining, their EVs components and biogenetic pathways are different [20,21]. In general, these bEVs are broadly categorized into outer membrane vesicles (OMVs) and cytoplasmic membrane vesicles (CMVs) based on their origin and secretion mechanisms (Figure 1) [22,23].

Over five decades of research have established that gram-negative bacteria employ three distinct vesiculation pathways, including OMVs [24], outer-inner membrane vesicles (OIMVs), and explosive outer membrane vesicles (EOMVs), produced multiple types of bEV under different conditions [25]. Most of the OMVs secreted by gram-negative bacteria are formed through a process called “blebbing,” resulting in vesicles being encapsulated in a single membrane bilayer, and the size of the bacterial OMV is similar to that of eukaryotic microvesicles and is caused by extrusion of the outer membrane [26]. OMV shedding can be attributed to a variety of mechanisms, including dissociation of stable cell wall crosslinking, reduction of outer membrane-peptide glycan linkage, increase of bacterial inner and outer membrane distance, increase of membrane curvature, local expansion and rupture of outer membrane, and orderly shedding of OMV regulated by bacterial genes, but the specific mechanism is still unclear [27]. In addition, a small number of gram-negative bacteria under genotoxic stress may be triggered by DNA damage or partial degradation of the peptidoglycan layer of the cell wall by autolysis to form pores, in which the inner and outer membranes protrude outward-wrapped cytoplasmic components to form double-layer vesicles with two membranes, and finally extrude from the bacterial surface to form OIMVs, resulting in death and lysis [28]. The membrane fragments produced by explosive cleavage can reassemble and randomly wrap the cytoplasmic components forming vesicles called EOMVs [29]. Notably, both OIMVs and EOMVs are distinguished by their cytoplasmic cargo enrichment, suggesting roles in bacterial stress response and horizontal gene transfer [30]. Although the cell wall of gram-positive bacteria lacks the outer membrane structure and is surrounded by a dense peptidoglycan layer, the current study believes that the weakening of the peptidoglycan layer by cell wall degrading enzymes and the increase of bacterial internal pressure led to the release of the bacterial inner membrane, and the bacterial plasma membrane wraps the cytoplasmic components and bulges outward. These reasons lead to the formation of CMVs. In addition, explosive CMVs (ECMVs) can be formed in gram-positive bacteria via “bubbling cell death,” which is similar to EOMV biogenesis. However, precise regulatory mechanisms governing CMV biogenesis remain elusive. While the specific mechanisms of different bEV production are still being actively investigated, both gram-negative and gram-positive bacteria include two major pathways of bubbling and explosive cell lysis [23]. A 2024 study by Juodeikis et al. revealed growth phase-dependent lipoprotein loading in B. thetaiotaomicron bEVs, with selective enrichment of negatively charged lipoproteins containing signaling peptides during late exponential phase, highlighting dynamic cargo regulation [31].

bEV is secreted from the surface of bacteria. Common methods for the extraction and purification of bEVs include ultrafiltration, density gradient centrifugation, ultracentrifugation, particle size exclusion chromatography, immunomagnetic bead separation, microfluidics, and aptamer-based magnetic separation [32]. Among them, density gradient centrifugation and ultracentrifugation are the “gold standards” for bEV separation [33]. Usually, a combination of ultracentrifugation and density gradient centrifugation is adopted. By taking advantage of the differences in size, density, and molecular weight between bEV and other biomolecules, high-purity bEV is obtained through long-term centrifugation and the addition of specific separation media. Because this method can reduce the damage of centrifugal force to bEV and has high separation purity, it is suitable for the functional research, content analysis, and biomarker detection of bEV [34]. At present, there are many advanced techniques available for analyzing bEV, including AIEgens, single-vesicle analysis, bEV-specific flow cytometry, and omics methods [35]. Single-vesicle analysis involves immobilizing bEVs in a microfluidic room, performing immunostaining and imaging. bEV-specific flow cytometry involves labeling them with fluorescent dyes or surface label-specific antibodies, thereby conducting quantitative evaluations based on fluorescence intensity. Omics methods can conduct comprehensive studies on the structure and function of proteins and genes in bEV. Depending on the different structures of bEV membranes, the regulated AIEgens can selectively illuminate bEVs without additional washing steps, and the accompanying fluorescence signals can further effectively achieve the identification and classification of bEVs [36].

The structure and content of the many types of bEV mentioned above depend on how they are produced and secreted. bEV production is dynamically regulated by intrinsic bacterial factors (adhesion machinery, antibiotic resistance genes) and extrinsic parameters including host age, diet, gastrointestinal physiology, and microbial community structure [37–41]. Importantly, vesicle composition serves as a molecular fingerprint reflecting bacterial physiological status, enabling subtyping of bEVs for diagnostic applications [42]. By elucidating evolutionary conservation of vesiculation pathways, it is possible to identify novel bEV subtypes as biomarkers of bacterial pathogenesis, and develop bEV-based therapeutic platforms for microbiome modulation.

## Substances in bEVs

As nanoscale spherical transporters, bEVs feature a phospholipid bilayer encapsulating diverse bioactive components. Their outer membrane is uniquely characterized by the directional embedding of lipopolysaccharides (LPS) and outer membrane proteins [43], with structural and functional diversification across bEV subtypes dictated by the asymmetry and fluidity of bacterial membranes [44]. In different bacterial bEV subtypes, the protein sorting and proportion are different, and they contain DNA or RNA with different functions [45]. High-resolution proteomic studies have identified over 3,500 bEV-associated functional proteins [46,47]. It has been found that the key compositional features of the protein composition system include subtype specificity, functional diversity, and signal transduction networks: 1) Distinct bacterial sources and subtypes exhibit differential protein sorting mechanisms and proportional regulation, dynamically controlled by bacterial gene expression and environmental stress [48]. 2) Dominated by adhesion proteins and exotoxins, these functional protein clusters enhance bacterial survival through host colonization, competitor inhibition, immune evasion, and environmental resistance [49]. 3) Signal proteins with specialized amino acid sequences undergo strict membrane receptor recognition for targeted loading [50]. Their N-terminal signal peptides critically determine vesicle trafficking specificity [51]. Recent studies have found that multimodal genetic cargos of nucleic acid transport network, including functional DNA, mRNA, and non-coding RNAs [52–54]. Vesicle membranes confer resistance to DNase degradation, preserving DNA antigenic epitopes, which is called DNA protection of bEV [55]. mRNA translational activity of bEV is capable of horizontal transfer and heterologous translation in host cells. The sorting hypothesis of nucleic acid transport network has demonstrated that nucleic acids may enter vesicles via specific recognition sequences, though molecular mechanisms remain elusive [56,57]. The LPS-phospholipid composite matrix forms the vesicle scaffold, which is the membrane structural foundation of bEV. LPS, as a critical endotoxin, serves three biological roles, including membrane surface antigenic determinant, host cell adhesion molecule, and innate immune activation ligand [58,59]. Recent studies confirm significant heterogeneity in bEV nucleic acid payloads, suggesting cross-kingdom genetic regulation networks. Notably, 78% of bEV-associated proteins exhibit virulence factor characteristics, providing new perspectives for deciphering bacterial pathogenesis.

## bEVs in intestinal immune regulation

The intestinal barrier system, essential for maintaining gut homeostasis, comprises three interdependent layers: chemical, physical, and immune barriers [60–62]. The intestinal chemical barrier, which is composed of antimicrobial peptides (AMPs) and bile acids, this layer segregates gut microbiota from epithelial cells while selectively suppressing bacterial proliferation [63]. The intestinal epithelial barrier is a physical barrier that formed by a continuous epithelial cell layer interconnected via tight junction complexes (e.g. occludin, claudins, and zonula occludens proteins), this structure physically isolates the luminal environment from underlying tissues [64]. The intestinal immune barrier is located subjacent to the epithelium, this barrier involves specialized immune cells including macrophages, dendritic cells, and neutrophils that orchestrate mucosal immunity [65]. The latest research has found that the potential role of bacteria migrating from the oral cavity to the intestinal tract, forming an “oral-intestinal axis,” may play a synergistic role in the pathogenesis of autoimmune diseases [66].

**Figure 2.**
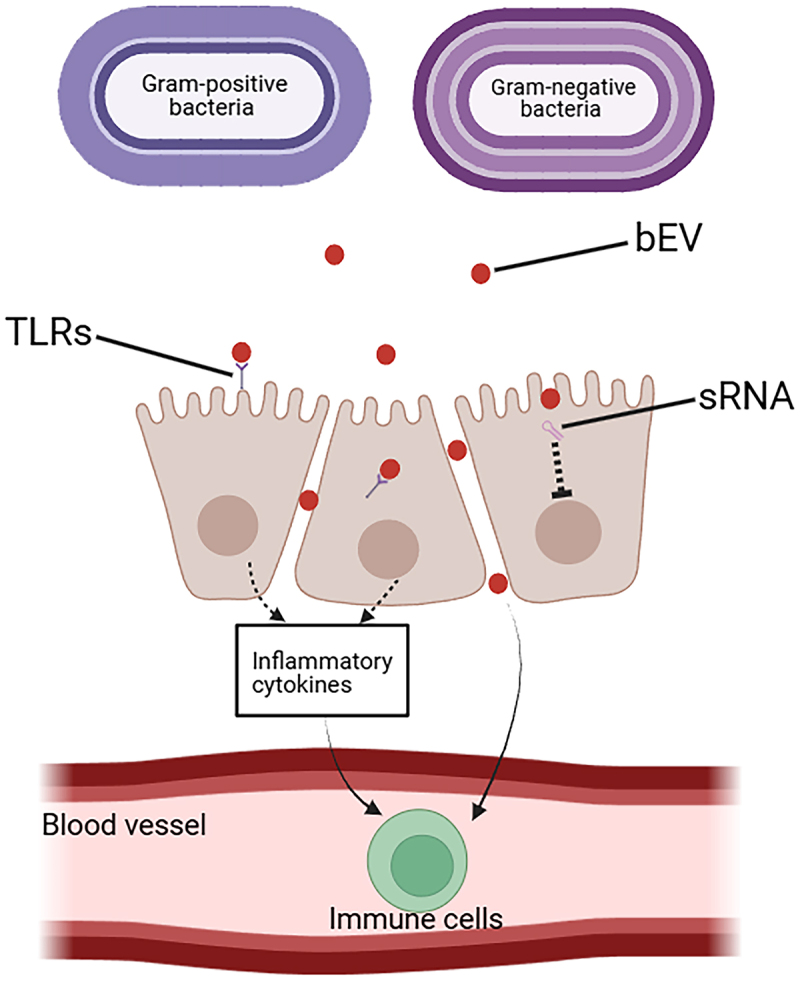
The regulations of bEVs in intestinal immune. Multiple cargoes carried by bEVs are recognized as pathogen-associated molecular patterns by toll-like receptors (TLRs) on the cell surface or endosome. This activation induces the release of cytokines that promote the inflammatory process. Some vesicles can penetrate the mucosal barrier through the paracellular pathway, enter the bloodstream and spread in the body. Some of bEVs may carry small RNAs (sRNAs) that bind to host mRNA to regulate their function.

Bacterial extracellular vesicles (bEVs) serve as critical mediators of host-microbe crosstalk (Figure 2). Studies have shown that bEVs from Escherichia coli (E. coli) strains can promote the production of IgG, IgA and IgM in the plasma of healthy lactating rats and induce a larger proportion of Tc, NK and NKT cells in the spleen. This indicates that bEVs can induce an innate immune response and be recognized by antigen-presenting cells, thereby initiating an adaptive immune response [67]. Fusobacterium nucleatum (F. nucleatum)-derived bEVs activate NF-κB, ERK, and CREB signaling pathways in epithelial cells, upregulating pro-inflammatory cytokines (TNF-α, IFN-γ, IL-6, MCP-1), reflecting the pathogen-specific activation of bEV [68]. Gram-Differential Responses have been found by Thapa et al. who comparative analysis of 32 bacterial strains (26 Gram-negative vs. 6 Gram-positive) revealed stronger pro-inflammatory effects from Gram-negative bEVs, particularly elevating IL-8, CCL20, and CXCL1 levels [69]. On the contrary, The EVs of Propionibacterium freudenreichii contains surface-layer protein B (SlpB), which proves its importance in regulating the immune response by preventing the release of IL-8 caused by LPS [70]. Another study showed receptor-specific signaling through Enterohemorrhagic E. coli (EHEC) O157 bEVs engage both surface (TLR4/5) and intracellular receptors (NOD1), activating NF-κB through distinct pathways [71]. Notably, peptidoglycan delivery via bEVs triggers NOD1-dependent innate immunity independent of TLRs [72]. One of the primary mechanisms studied for immune activation of bEV may be the activation of TLRs or other pathogenrecognition receptors (PRRs), which recognize pathogen-associated molecular patterns (PAMPs) to promote immune response [73]. In fact, because of bEVs carry different cargo, they can activate different TLRs with different strengths. Helicobacter pylori (H. pylori) bEVs demonstrate strain-specific activation of endosomal TLR7 (RNA-mediated) and TLR9 (DNA-mediated) [74]. However, Staphylococcus aureus (S. aureus) bEVs exhibit preferential TLR2 activation via lipoproteins, contrasting with NOD2/TLR7/8/9 activation by peptidoglycans [75]. This suggests that Gram-positive bacteria can also carry lipoproteins, peptidoglycan, and nucleic acids recognized by host PRRs and are characterized by cargo-dependent activation. In contrast, probiotic E. coli Nissle bEVs require clathrin-mediated endocytosis and NOD1 recognition to stimulate IL-8/IL-6 secretion [76,77]. Clostridial bEVs differentially induce CCL2/IL-6 in Caco-2 versus CXCL2 in CMT-93 cells, highlighting epithelial heterogeneity [78]. This suggests that bEV promotes the expression of different inflammatory mediators depending on which cells they interact with, a phenomenon that reflects the cell-type specific effects of bEV. bEVs exhibit paradoxical effects on intestinal barriers, playing a dual role in barrier function (Table 1) [81,82]. Its destructive potential is manifested in its capable of breaching physical barriers through enzymatic activity (e.g. nutrient acquisition enzymes) and neutralizing host defenses (antibodies, antibiotics) [83]. This immune regulation of bEV may help protect Pseudomonas aeruginosa (P. aeruginosa) from the immune system as it grows [84]. Of the thousands of unique small RNA (sRNA) sequences that bEVs from P. aeruginosa may carry, some are predicted to bind to human mRNA and thus affect the immune response. The question is whether the presence of these bacterial sRNA in bEVs also influences the function of the host cells that internalize these vesicles, as do key mechanisms in the regulation of bacteria [85]. Interestingly, bEVs secreted by Pseudomonas gingivalis (P. gingivalis), which can transport bacterial DNA and related effect molecules, may induce systemic inflammation and disrupt the intestinal barrier, promoting the leakage of intestinal substances into the bloodstream [86]. Facilitate interkingdom communication by transporting immunomodulatory molecules, maintaining gut microenvironment equilibrium through local and systemic signaling, which reflects the protective function of bEVs [5,87]. Gram-differential immunomodulatory mechanisms: Gram-negative bEVs primarily activate surface TLRs (e.g. TLR4/5) and cytosolic sensors (NOD1) via LPS/peptidoglycan, driving robust IL-8-dominated inflammation [69,71]. Gram-positive bEVs rely on lipoprotein-TLR2 and peptidoglycan-NOD2 signaling but exhibit attenuated cytokine responses due to structural differences in PAMPs [75,78]. Endothelial crossing: bEVs exploit transcytosis or inflammation-induced barrier breach to enter systemic circulation, facilitated by membrane fluidity and pathogen-derived proteases [7,88].

**Table 1.** Comparative effects of commensal vs. pathogenic bEVs on immunity.

Feature	Commensal-Derived bEVs	Pathogen-Derived bEVs
Key Components	Polysaccharides, anti-inflammatory miRNAs	Toxins (e.g. CNF1, VacA), virulence factors
TLR Activation	TLR2/TLR4 (moderate; promotes Tregs)	Cytokine Profile: ↑ IL-10, TGF-β; ↓ TNF-α
Cytokine Profile	↑ IL-10, TGF-β; ↓ TNF-α, IL-6	↑ IL-1β, IL-8, TNF-α; ↓ IL-10
Barrier Function	Enhance epithelial integrity	Disrupt tight junctions (e.g. via proteases)
Immune Cell Targets	Dendritic cells → Treg differentiation	Macrophages → pyroptosis; Neutrophils → NETosis
Clinical Association	Protection in colitis (e.g. A. muciniphila)	Sepsis, IBD exacerbation (e.g. EHEC)
References	[77,79]	[71,80]

Summarizing, the dual nature of bEVs – acting as both disruptors and regulators of intestinal barriers – underscores their pivotal role in gut homeostasis. These findings illuminate the sophisticated dialogue between gut microbiota and host immunity, providing mechanistic insights into microbial contributions to health and disease pathogenesis.

## bEVs and macrophage-mediated inflammation

**Figure 3.**
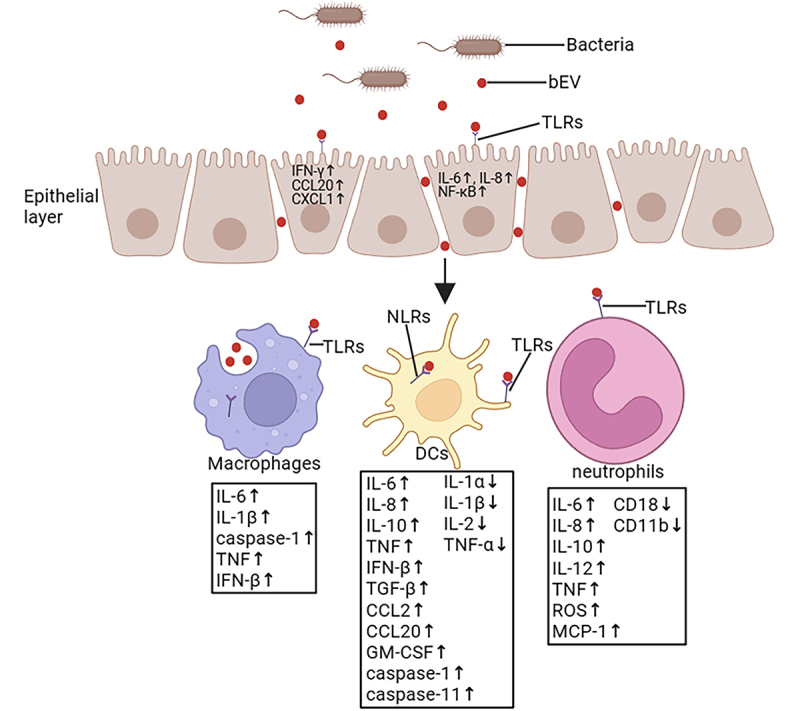
The functions of bEVs with intestinal immune cells. Some of the bEVs secreted and released by bacteria are absorbed by epithelial cells and increase their cytokines release, and some are captured by immune cells after passing gut vascular barrier (GVB). The multiple cargo carried by bEVs is recognized by cell surface receptors (such as TLRs) and intracellular receptors (such as NODs) as pathogen-associated molecular patterns (PPRs). This activation induces the release of cytokines that promote inflammatory processes, such as the secretion of IL-8, and promotes recruitment and activity of neutrophils.

As key effectors of innate immunity, macrophages actively internalize bacterial extracellular vesicles (bEVs) that breach mucosal barriers. Similar to epithelial cells, macrophages respond to bEVs through TLR activation, triggering inflammatory mediator release [89]. Similar to epithelial cells, macrophages respond to bEVs through TLR activation, triggering inflammatory mediator release (Table 2). These nanoscale carriers can initiate multiple pro-inflammatory cascades via surface recognition or phagocytic uptake (Figure 3). Kim et al. demonstrated that pathogen-derived bEVs frequently dysregulate host immune responses [80] – a prime example being E. coli bEVs delivering virulence factors to intestinal macrophages, driving TNF-α and IL-6 overexpression linked to systemic inflammatory response syndrome (SIRS) and sepsis. Notably, the heat-labile enterotoxin (LT) on E. coli bEVs directly engages PRRs on macrophages to activate pro-inflammatory signaling and chemokine production [90,106,107]. Plasmid-encoded HlyF exemplifies another virulence strategy, enhancing bEV biogenesis in E. coli [108]. David et al. revealed that HlyF-positive E. coli bEVs induce gasdermin-D-dependent pyroptosis via non-canonical inflammasome activation, amplifying IL-1β secretion and macrophage cytotoxicity [91]. Mitochondrial dysfunction emerges as a pathological mechanism, as evidenced by Acinetobacter baumannii (A. baumannii) bEVs carrying OmpA toxin disrupting mitochondrial dynamics in murine macrophages [92]. Recent studies have found that flagellated pathogens exhibit distinct delivery mechanisms. Pseudomonas aeruginosa (P. aeruginosa) and Salmonella typhimurium (S. typhimurium) bEVs exploit endocytosis to activate NLRC4 inflammasomes, promoting caspase-1 (cysteine-aspartic protease)-dependent IL-1β maturation [93]. Clostridial species employ TLR2-dependent pathways in RAW264.7 macrophages to upregulate TNF, IL-6, and IL-1β, with vesicle internalization requiring actin polymerization and partially relying on PI3K/MyD88 signaling [78]. Intriguingly, TLR inhibition incompletely suppresses cytokine release, implying nucleic acid components may synergize with other PRR ligands in immune activation. Other studies have shown that Staphylococcal bEVs demonstrate paradoxical immunomodulation. S. aureus-derived vesicles stimulate IFN-β expression in NR-9456 macrophages through endosomal TLR3/7/9 activation, balancing pro-inflammatory and immunosuppressive effects [94,109]. Anthrax pathogenesis involves toxin-laden vesicles – Bacillus anthracis (B. anthracis) bEVs concentrate edema and lethal toxins, inducing macrophage cytolysis through undefined mechanisms [95]. Even commensal-derived vesicles may trigger pathology, as shown by Bacteroides thetaiotaomicron (B. thetaiotaomicron) bEVs promoting colitis in susceptible mice via macrophage-targeted delivery, though precise pathways remain elusive [96]. Collectively, these findings underscore the pleiotropic nature of bEV-mediated inflammation – diverse cargoes (toxins, PRR ligands, nucleic acids) engage multiple receptors (TLRs, NLRs, inflammasomes) through varied entry mechanisms (endocytosis, membrane fusion, phagocytosis). This functional redundancy highlights evolutionary optimization for immune subversion, as pathogens exploit bEVs to attenuate macrophage defenses. While mechanistic details require further elucidation, the emerging paradigm positions bEVs as critical mediators of microbiota-immune crosstalk, with implications for infectious diseases and inflammatory disorders.

**Table 2.** The function of bEVs in immune cells.

Cell types	Bacteria	Effective contents	Molecules	Influences	Reference
Macrophages	E. coli	Virulence factors	TNF-α↑ and IL-6↑	SIRS and sepsis	[90]
	E. coli	HlyF	IL-1β↑	macrophages death	[91]
	A. baumannii	OmpA	NA	Disrupted the mitochondrial morphology	[92]
	p. aeruginosa and S. typhimurium	NLRC4	caspase-1↑ and IL-1β↑	Inflammation	[93]
	Clostridium	PI3K and MyD88	TNF↑, IL-6↑ and IL-1β↑	Inflammation	[78]
	S. aureus	NA	IFN-β↑	Inflammation	[94]
	B. anthracis	edema toxins and lethal toxins	NA	Anthrax	[95]
	B. thetaiotaomicron	NA	NA	Colitis	[96]
DCs	Bacteroides tenuis	Gadd45α	IL-10↑	anti-inflammation	[77]
	L. paracasei	COX-2, iNOS, NF-κB and NO	TNF-α↓, IL-1α↓, IL-1β↓, IL-2↓, TGF-β↑ and IL-10↑	anti-inflammation	[97]
	A. muciniphila	NA	NA	Relieve colitis	[79]
	V. cholerae		GM-CSF↑, IL-8↑, CCL20↑ and CCL2↑	T-cell polarization	[98]
	H. pylori	VacA	IL-10↑ and IL-6↑	T cell apoptosis	[99]
Neutrophils	H. pylori	LPS	IL-8↑	sepsis	[100,101]
	NA	MyD88	IL-6↑, TNF-α↑, MCP-1↑, ROS↑ and IL-10↑	Inflammation	[102]
	S. aureus	NA	TNF↑, IL-12↑ and IL-6↑	Neutrophils polarization	[103]
	E. coli	CNF1	CD18↓ and CD11b↓	Reduce the phagocytosis and chemotaxis of neutrophils	[104,105]

NA: No accessible data in the study.

Taken together, bEVs drive context-specific inflammation via TLR/NLR ligation and toxin delivery, with pathogen-derived vesicles promoting cytotoxicity (e.g. E. coli HlyF-induced pyroptosis) and commensal vesicles resolving inflammation [77,91].

## bEVs and dendritic cells with immunomodulatory effects

bEVs activate dendritic cells (DCs) through pathogen-associated molecular patterns (PAMPs), which include surface-exposed molecules like lipopolysaccharides (LPS) and peptidoglycans, as well as intracellular components such as bacterial DNA (Table 2). These PAMPs are recognized by DCs via distinct pattern recognition receptors (PRRs) (Figure 3): Membrane-bound PRRs are recognized by DCs via Toll-like receptors (TLRs, e.g. TLR4 for LPS recognition) on the cell surface [110]. In contrast, Caspase family proteins (e.g. Caspase-11) that detect cytosolic LPS and trigger inflammatory cascades [81]. Nucleotide-binding oligomerization domain (NOD)-like receptors (NLRs, e.g. NOD1/2) that sense peptidoglycan derivatives [111]. The above two parts together constitute cytoplasmic PRRs. bEVs derived from probiotics have mostly been shown to have beneficial effects on host immune regulation. For example, commensal bacterial EVs generally exhibit immunomodulatory benefits. Bacteroides thetaiotaomicron bEVs carry surface polysaccharides that promote IL-10 production in DCs and regulatory T cells via TLR2/Gadd45α signaling and demonstrate protective effects in murine acute colitis models by maintaining intestinal homeostasis [77]. Lactobacillus paracasei (L. paracasei) bEVs suppress NF-κB-mediated inflammatory pathways, downregulating COX-2, iNOS, and pro-inflammatory cytokines (TNF-α, IL-1β), enhance anti-inflammatory mediators (TGF-β, IL-10), mitigating LPS-induced colonic inflammation, and oral administration alleviates dextran sulfate sodium (DSS)-induced colitis symptoms, preserving colon length and body weight [97]. Similarly, bEVs secreted by Akkermansia muciniphila (A. muciniphila) has been shown to attenuate DSS-induced colitis severity through immune barrier reinforcement and inflammatory pathway modulation [79]. In contrast, pathogenic bacteria often release pathogen-derived bEVs in immune dysregulation to disrupt immune homeostasis [80]. For example, Vibrio cholerae (V. cholerae) O395 bEVs enter DCs via porin-mediated endocytosis and induce GM-CSF, IL-8, CCL20, and CCL2 secretion, driving Th2/Th17 polarization and pro-inflammatory responses [98,112]. In the case of Helicobacter pylori (H. pylori), bEVs deliver vacuolating cytotoxin A (VacA) to monocytes, elevating IL-6/IL-10 while suppressing CD4+ T cell proliferation and promote T cell apoptosis, facilitating immune evasion [99]. When bEVs recognize and bind to DCs, bEV-DC interactions have a dual role, whether they benefit host immune regulation depends on the source of the bacteria and the substances they carry [113,114]. bEVs have immunostimulatory effects: surface PAMPs (LPS/peptidoglycan) activate DCs to sustain baseline immune vigilance. However, this process may lead to immunopathological risks. Overactivation may lead to immunosuppression, cytokine storms, or immune tolerance [115,116]. Intracellular delivery of virulence factors (e.g. sDNA, toxins) can reprogram DC function, creating permissive conditions for bacterial invasion [117].

In summary, DC responses are cargo- and source-dependent: probiotic bEVs (e.g. B. thetaiotaomicron, L. paracasei) induce anti-inflammatory IL-10/TGF-β, while pathogenic bEVs (e.g. V. cholerae, H. pylori) drive Th17 polarization and immune evasion [77,97–99].

## A bidirectional immunomodulatory relationship between bEVs and neutrophils

The interaction between gastric epithelial cells and Helicobacter pylori (H. pylori)-derived bEVs represents the earliest identified mechanism linking bacterial vesicles to neutrophil-mediated inflammation (Table 2). Recent studies have found that bEVs from Parabacteroides goldsteinii (P. goldsteinii) of the intestine can activate the Cav-1-Nrf2 axis, leading to a reduction in the formation of neutrophil extracellular traps and alleviating inflammation [118]. Studies demonstrate that H. pylori bEVs activate the LPS receptor TLR4 on posterior gastric epithelial cells, triggering IL-8 secretion and subsequent neutrophil recruitment through MyD88-dependent signaling [100]. While this chemokine-driven neutrophil influx serves as a frontline defense against infection, emerging evidence reveals bEVs possess dual capacity to either amplify or attenuate inflammatory responses through direct neutrophil modulation (Figure 3). Experimental models using Escherichia coli (E. coli) bEVs highlight the dichotomy of dose-dependent immunomodulation by bEVs [119]: intraperitoneal administration (50 μg bEV protein) induces systemic neutrophil infiltration accompanied by sepsis-like pathology, whereas lower doses exhibit priming effects [101,120]. Low-dose priming: enhances pro-inflammatory sensitivity through elevated IL-6, TNF-α, MCP-1, and reactive oxygen species (ROS) production, coupled with increased phagocytic activity [121–123]. In contrast, high-dose exposure: induces immune tolerance characterized by IL-10 upregulation and suppressed neutrophil migration/phagocytosis [102,124,125]. Mechanistically, these dose-responsive effects correlate with differential activation of TLR4/MyD88 and TLR2/MyD88 pathways in neutrophils. Kim et al. demonstrated that S. aureus bEV Drive Th1/Th17 polarization through neutrophil-derived IL-6, IL-12 and TNF production [103]. In addition to recruiting them, E. coli bEV has also been shown to regulate the antimicrobial activity of neutrophils. Cytotoxic E. coli strains utilize bEVs as toxin-delivery vehicles carrying CNF1, which subverts neutrophil function via rho GTPase-mediated disruption of actin polymerization [104], downregulation of CD18/CD11b complement receptors [105], and impaired chemotaxis and phagocytic capacity. These findings establish gut microbiota-derived bEVs as critical regulators of neutrophil plasticity through multiple mechanisms. BEVs have become a direct receptor-mediated signal and could have interesting therapeutic implications through epigenetic reprogramming. bEV also plays an important role in cytokine network modulation. The dual pro-/anti-inflammatory potential of bEVs underscores their therapeutic relevance in inflammatory disorders and antimicrobial strategies, warranting further investigation into their spatiotemporal regulation of neutrophil responses.

To summarize, bEVs exhibit dose-dependent duality – low doses prime neutrophil activity (ROS/phagocytosis), while high doses induce tolerance (IL-10 ↑, migration ↓). Pathogen-derived vesicles (e.g. E. coli CNF1) directly subvert antimicrobial functions [102,104,119].

## Related diseases and therapeutic applications

Given the significant role of bEVs in maintaining intestinal homeostasis, we can appropriately study their possible involvement in the occurrence and progression of intestinal-related diseases. Current research indicates that bEV plays a key role in various intestinal-related diseases, including infections, inflammatory bowel disease (IBD), and cancer. Many studies have discovered the role of bEV in infecting and harming the host. bEVs can promote the formation of biofilms and complex microbial communities [25,126]. In addition, bEVs can neutralize the activation of AMP, potentially disrupt the effectiveness of the host’s chemical barrier and enhance its susceptibility to pathogenic infections, which are key factors for gastrointestinal infections [127,128]. A large number of researches have shown that intestinal barrier dysfunction can accelerate the progression of IBD. The increased proportion of Gram-negative bacteria observed in IBD patients usually releases excessive bEV loaded with LPS. These bEVs infiltrate epithelial cells, and their LPS are transported to the cytoplasm, triggering an immune response, down-regulating E-cadherin expression, and causing intestinal barrier dysfunction [129]. Current studies indicate that bEV leads to IBD by disrupting the physical and immune barriers composed of epithelial cells and immune cells. A large number of reports have confirmed that bEVs have a significant impact on the occurrence and development of gastrointestinal cancers. Studies have shown that bEVs of E. coli MG1655 can deliver a tRNA fragment Ile-tRF-5X to human colorectal cancer cells. Activate mitogen-activated protein kinase 3 (MAPK3), thereby promoting cell proliferation [130]. F. nucleatum, a pathogen causing colorectal cancer, functions through multiple mechanisms including bEVs. Mass spectrometry analysis revealed that these bEVs exhibited a selective enrichment phenomenon, with a large number of virulence factors and bioactive proteases present [131]. The specific roles of bEV in colorectal cancer include: simultaneously reducing the gene expressions of E-cadherin and cadherin-1 and promoting genotype alterations similar to epithelial-mesenchymal transition in tumor cells [132]; inducing IL-8 expression and forming a pro-inflammatory microenvironment conducive to tumor growth [133], these factors act together and ultimately promote the migration and invasion of cancer cells in the body [134].

Due to the complex interaction between bEV and host cells, it may play a key role in the pathogenesis of diseases. Therefore, bEV can be applied in the treatment of diseases. bEVs demonstrate significant diagnostic potential, where 16S rRNA profiling effectively discriminates Crohn’s disease from ulcerative colitis, enabling noninvasive IBD diagnostics [135], while AIEgen-based tracking facilitates real-time visualization of bEV dynamics during intestinal barrier dysfunction [36]. Therapeutically, engineered E. coli bEVs deliver osteogenic miRNAs for osteoporosis treatment [136], L. murinus-derived vesicles attenuate intestinal inflammation through tight junction reinforcement [137], and tumor-targeting bEVs suppress progression in bone/soft tissue malignancies [138]. However, clinical translation faces critical challenges: standardized isolation protocols (e.g. MISEV-compliant methodologies) and scalable production systems remain essential for deployment [139], alongside unresolved safety concerns including dose-dependent inflammatory responses and horizontal gene transfer risks.

## Conclusion and future prospects

The gut microbiota plays a pivotal role in modulating human health and disease pathogenesis. Bacterial extracellular vesicles (bEVs), nanosized particles secreted by gut microbes, carry a diverse cargo of bioactive molecules such as lipopolysaccharides, peptidoglycans, proteins, nucleic acids, and virulence factors. These components enable bEVs to participate in critical biological processes including bacterial communication, antibiotic resistance, host-microbe interactions, and immune regulation. Over the past decade, bEV research has advanced significantly, revealing their therapeutic potential in maintaining intestinal homeostasis, serving as drug delivery vehicles, and functioning as immune modulators. Current applications predominantly focus on vaccine development, targeted therapies, and diagnostic biomarker discovery. Notably, emerging evidence demonstrates growth phase-dependent variations in bEV composition, with selective enrichment of negatively charged lipoproteins containing signaling peptides in outer membrane vesicles (OMVs) [31]. These findings provide novel insights into bEV biogenesis and release dynamics.

Critical unresolved questions persist regarding whether bEVs from dysbiotic microbiota contribute to extraintestinal pathologies and if engineered bEVs can overcome biological barriers for targeted drug delivery. Addressing these requires prioritized clinical initiatives: 1) establishment of standardized isolation protocols and reference bEV panels; 2) development of engineered bEV platforms with tunable immunogenicity; and 3) implementation of advanced technologies like AIEgen-based in vivo tracking and single-vesicle omics [36]. Key advances illuminate promising pathways – temporal lipoprotein loading in B. thetaiotaomicron bEVs [31] and transcytosis/inflammation-dependent gut-vascular barrier (GVB) crossing [88] – while therapeutic innovations demonstrate efficacy in inflammation resolution (L. murinus bEVs) and osteoporosis treatment (engineered bEVs) [136,137]. Nevertheless, significant challenges remain, including incomplete understanding of Gram-positive biogenesis regulatory mechanisms, lack of real-time trafficking models, scalability limitations in therapeutic production, safety profiling demands, and the need for single-vesicle functional analysis of diagnostic technologies like AIEgen tracking and 16S-rRNA profiling [36,135]. Future research must bridge these gaps to harness the full diagnostic and therapeutic potential of bEVs.

## Data Availability

No new data are included in this article. Data in referenced prior studies may be accessible in the original works.
